# Resistance to the Whitefly, *Aleurotrachelus socialis*, in Wild Populations of Cassava, Manihot Tristis

**DOI:** 10.1673/031.010.14130

**Published:** 2010-10-05

**Authors:** A. Carabalí, A. C. Bellotti, J. Montoya-Lerma, Martin Fregene

**Affiliations:** ^1^ Centro Internacional de Agricultura Tropical (CIAT), A.A. 6713, Cali, Colombia; ^2^ Grupo de Investigaciones Entomológicas, Departamento de Biología, Universidad del Valle, AA 25360, Cali, Colombia

**Keywords:** antixenosis, wild species

## Abstract

The levels of resistance in the wild species of cassava, *Manihot tristis* Muell-Arg. (Malpighiales: Euphorbiaceae), to the whitefly, *Aleurotrachelus socialis* Bondar (Hemiptera: Alelyrodidae), the most important economic pest in cassava, *Manihot esculenta* Crantz (Malpighiales: Euphorbiaceae) crops in South America, were estimated under glasshouse conditions. The parameters of the life history of *A. socialis* were studied on TST-26 and TST-18 accessions of the wild parent and compared with the susceptible (CMC-40) and resistant (MEcu-72) cultivars. The average longevity on the wild accessions (TST-26, 4.1; TST-18, 4.6 days) and oviposition rates (TST-26, 2.0; TST-18, 1.6 eggs/female/2 days) of the *A. socialis* females were not significantly different from those of MEcu-72 (5.1 days and 3.4 eggs/female/2days). The longevity and oviposition rates on CMC-40 were highest (11 days and 8.6 eggs/female/2days). Analyses of the demographic parameters (Ro, r_m_; DT) showed a significant impact of the *M. tristis* accessions on the potential growth of *A. socialis.* The average survival time of adults that fed on TST-26, TST-18, and MEcu-72 were significantly different from those recorded on the susceptible genotype. Results from this study revealed important levels of resistance to the whitefly *A. socialis* on the TST-26 and TST-18 accessions due to the marked differences found for longevity and reproduction, which influenced and were consistent with the differences found in the net reproduction rate (Ro), intrinsic growth rate (r_m_) and population doubling time (DT). The combined effect of these parameters indicated that *M. tristis* accessions were inappropriate hosts for *A. socialis.*

## Introduction

The *Manihot* genus belongs to the Euphorbiaceae family, Crotonoideae subfamily and Manihotae tribe and includes approximately 100 species of herbs, bushes and trees, with the shared characteristic of producing latex and cyanogenic glucosides (Bailey 1976). Cassava, *Manihot esculenta* Crantz (Malpighiales: Euphorbiaceae) is a perennial bush from South America and is a basic staple for a great number of countries in Africa, Asia, and Latin America (FAO/FIDA 2000). Although the roots are a source of carbohydrates in the human diet, *M. esculenta* is also widely used as a raw material in the processed products industry. In addition to the economic advantages that its products and byproducts offer, *M. esculenta* is a crop that grows well under marginal conditions. In general, its varieties are tolerant to drought, and grow and produce well in degraded soils (Ceballos 2002).

Given its great agro-ecological diversity, Colombia has a wide range of planting systems, from dry and semiarid regions, passing through inter-Andean valleys and the flatlands of the Eastern Plains, to the rainy Pacific Coast region (Balcazar 1997). This diversity of environments brings with it a wide range of biological problems, including diseases and arthropod pests, the majority of which are endemic (Henry and Hershey 2002).

It is estimated that 200 species of arthropod pests are associated with *M. esculenta* (Bellotti and Van Schoonhoven 1978) many of which are specific and/or adapted, in various forms, to the natural biochemical defenses of the plant that include laticiferous and cyanogenic compounds (Bellotti and Riis 1994). It is thought that these pests have coevolved with the crop (Bellotti et al. 1999; Bellotti 2002), among which the following are most important: the mite, *Mononychellus tanajoa,* the mealybug, *Phenacoccus herrenii,* the hornworm *Erinnyis ello,* the stemborer, *Chilomima clarkei,* the fruit fly, *Anastrepha manihoti,* the thrips, *Scirtothrips manihoti,* and the whiteflies, *Bemisia tabaci, Aleurotrachelus socialis,* and *Aleurothrixus aepim.* Field research indicates that extended attacks (3–6 months) by mites, mealybugs, thrips, and whiteflies can cause up to a 79% loss in root yields (Bellotti et al. 1999). The whiteflies are the most important *M. esculenta* crop pests in the Americas, Africa, and to a lesser extent, Asia because of their proven efficiency as vectors of viruses as well as the damage caused by their direct feeding and excretion of honey dew (Brown et al. 1995; Oliveira et al. 2001). To date, *A. socialis* Bondar (Hemiptera: Alelyrodidae) appears to be specific to *M. esculenta* and is the predominant species in the northern part of South America (Venezuela, Panama, Ecuador, and Colombia) and, to a lesser extent, in Brazil (Farias 1994). In Colombia, it is the species of greatest economic importance causing losses of up to 79% in root yield (Bellotti and Arias 2001; Vargas and Bellotti 1981). Over the last five years, the *A. socialis* populations have increased and are endemic in the provinces of Cauca, Quindio, and Valle del Cauca (Colombia) (Bellotti 2002).

In the Neo-tropics, *M. esculenta* crops are exposed to the pressure of *A. socialis* populations for a long period of time (8–24 months), and traditional control measures based on the use of costly insecticides make the crop unprofitable. Alternative control measures emphasize cultural practices, the use of natural enemies, and host plant resistance (CIAT 2006). Although this last alternative is not frequent, promising sources of resistance have been identified and incorporated into productive hybrids (Bellotti 2002). The importance of this option lies in its being a rational, easy-to-adopt practice for keeping *A. socialis* populations low and reducing yield losses (CIAT 2004). The wild parents of M *esculenta* are known sources of genes resistant to insect pests. For example, *Manihot peruviana* and *Manihot flabellifolia* have shown from moderate-to-high levels of resistance to the cassava green mite. *Mononychellus tanajoa* and *A. socialis,* respectively (Burbano et al. 2003).

The purpose of these studies was to quantify levels of resistance to the whitefly *A. socialis* of two accessions of the wild species *Manihot tristis* Muell-Arg. (Malpighiales: Euphorbiaceae) based on biological and demographic parameters of the whitefly's life history.

## Materials and Methods

### Plants, insects, and environmental conditions

Host plant resistance studies, initiated at Centro Internacional de Agricultura Tropical (CIAT) more than 15 years ago, have systematically evaluated nearly 6000 accessions from the CIAT's cassava germplasm bank. Results identified sources of resistance to *A. socialis.* Cultivar MEcu72 consistently has, expressed the highest level of resistance (Bellotti and Arias 2001), while CMC-40 has been identified as the most susceptible. *A. socialis* colonies are maintained on this cultivar. Field evaluations of eight *Manihot* species, 22 accessions, and studies of oviposition preference showed that *A. socialis* feeding on *M. tristis* TST-18 and TST-26 accessions had lower population levels and less oviposition (CIAT 2006). In this study, the experiments included two accessions (TST-26 and TST-18) of *M. tristis,* the wild parent and a potential source of resistance to *A. socialis*; and the cultivars MEcu-72 and CMC-40of *M. esculenta,* with high resistance (Bellotti and Arias 2001) and susceptibility (Holguín et al. 2006), respectively, to the whitefly,. For each genotype (CIAT Cassava Genetics Program), 30 seedlings were established *in vitro* from embryo axes, multiplied, and then planted in sterile soil in 1-kg plastic pots. The plants did not receive pesticide or fertilizer applications. The *A. socialis* adults used in the trials were taken from the colony established at CIAT in 1992 on plants of the susceptible CMC-40 cultivar. Potted cassava plants produce about 20,000 *A. socialis* adults daily (Bellotti and Arias 2001). All experiments were conducted in a glasshouse at a mean temperature of 25° C (± 5° C) and an average RH of 70% (range: 60–90%). These studies were conducted at CIAT (Palmira, Colombia) in 2007.

### Longevity and fecundity

From the *A. socialis* colony, 40 recently emerged pairs (male: female) were selected and placed in separate clip cages (diameter = 2.5 cm; height = 2 cm) and given a number from 1 to 4 with the aid of a manual aspirator (that during use became coated with wax from the wings of adults which reduced mortality during handling). The adults were placed on the underside of the youngest leaves of 40-day-old TST-26, TST-18, CMC-40, and MEcu-72 plants. Twenty plants per genotype with two clip cages per plant were arranged randomly. The experimental unit consisted of a single leaf with a caged pair of whiteflies. Every 48 h, adults were moved to a new leaf in new leaf cages. The leaf portion under each cage was marked with the number assigned at the beginning of the assay. This was repeated during the entire study until the females died. Males were replaced as they died but only until the fourth day of the assay. The leaf portion under each leaf cage was marked and observed under a stereo-microscope (40X) for the number of eggs laid. Fecundity was estimated as the number of eggs per female laid every 48 h, and longevity as the maximum time (days) that a female lived.

### Development time, survival rate, and proportion of females

Groups of 50, two-day-old adults (males and females) of *A. socialis* were placed in clip cages (diameter = 2.5 cm; height = 2 cm) on the underside of the leaves of TST-26, TST18, CMC-40, and MEcu-72. After 6 h the adults were removed, and 200 eggs from each lot were selected at random. Those remaining were removed with a needle and a fine brush. The evaluation of the development time, survival rate and proportion of females was made using a random design with ten plants per genotype. The experimental unit consisted of a plant with 200 eggs per genotype. Observations began at fifteen days post infestation, when the immature stages had developed, to determine the first day of adult emergence. The adults obtained daily were collected and the proportion of male to female recorded. Egg to adult development time was calculated by the formula:

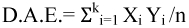

Where D.A.E. represents “days after emergence”, *X_i_* is the number of emerged adults at day *i*, *Y_i_* is the number of days from infestation to emergence of the adults at day *i*,and *n* is the total number of emerged adults.

The survival rate of immature individuals was determined using the relation between the number of *exuvia* (empty pupae capsules) and the number of eggs that were initially recorded. When the emergence of adults stopped, the leaves of each plant were cut and the number of empty pupae capsules were recorded under stereomicroscope. The survival rate was calculated using the formula

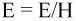

where, E is the number of empty pupae capsules per plant and H is the number of eggs per plant

### Demographic parameters

The experimental data on the development time of immature individuals and reproduction rates were combined to generate life tables (l_x-_m_x_) which were then used to calculate the demographic parameters (Price 1975): (1) Net reproduction rate (R_0_) or average number of descendents that a female produces in a generation; (2) generation time (T), which is equivalent to the period elapsed between the emergence of the parents and the emergence of their offspring, and (3) intrinsic growth rate of the population (r_m_), estimated using Carey's (1993) equation:

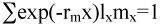

where, *X* = age of the female, l_x_ = specific survival age and m_x_ = the number of females in the progeny of a female at age x

Pivotal age, i.e. X + 0.5, was used to calculate r_m_ values, following Carey (1993). The formula In 2/r_m_ was used to estimate the number of days required for the population to double.

### Statistical analyses

To compare the female survival rates on the different host species, median survival times were calculated using the Kaplan-Meier test which includes the Gehan-Wilcoxon, Cox-Mantel, and Peto-Wilcoxon statistical tests (Lee 1992) (Statistix 8.0). Differences among the mean values for longevity, fecundity, female oviposition rate and development time (egg to adult) were analyzed using one-way ANOVA. Student-Newman-Keuls was used for the multiple-comparison tests. Survival rates and immature stage values were compared using the chi square test (SAS Institute 1989). Life table parameters were estimated using the jackknife technique, and the means were compared by *t*-test using the SAS LIFETABLE software developed by Mara et al. (2000).

## Results and Discussion

### Longevity and fecundity

**Figure 1.**
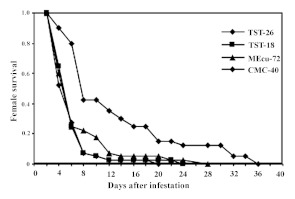
Survival curves of *Aleurotrachelus socialis* females on *Manihot tristis* accessions, resistant MEcu-72 and susceptible CMC-40 genotypes. High quality figures are available online.

The means of *A. socialis* survival time on the *M. tristis* TST-26 (10.1 days) and TST-18 (10.3 days) accessions and on the susceptible CMC-40 (14.9 days) and resistant MEcu-72 (10.5 days) cultivars are shown in Table 1. The results of all three survival analyses (Gehan-Wilcoxon, Cox-Mantel, and Peto-Wilcoxon) were consistent. The entries fell into two significantly different groups: one group containing TST-18, TST-26, and MEcu-72 with approximately 10-day longevity, and a second group containing only CMC-40 with 15-day longevity. All the *A. socialis* females used in the study survived the first 48 h on the genotypes evaluated, after which there was a decrease in their survival rate. These differences can be observed on the survival curves (Figure 1), where on day 10 the proportion of live females was reduced by 95, 95, 82 and 57%, respectively, on the *M. tristis,* MEcu-72, and CMC-40 accessions.

**Table 1.**
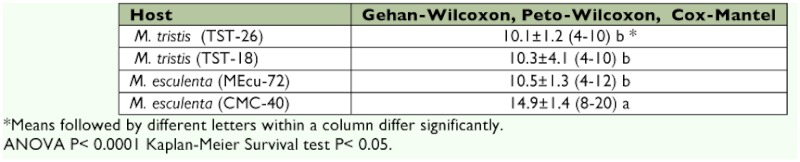
Mean survival time (± SE, days) test for female *Aleurotrachelus socialis* adults on *Manihot tristis* accessions, resistant MEcu-72 and susceptible CMC-40 (*n*=40) genotype.

Mean longevity was significantly greater on CMC-40 than on the other three hosts (approximately six days longer). There were no significant differences among TST-18, TST-26, and MEcu-72.

The mean fecundity of the *A. socialis* females on the different hosts examined had a broad range of 10–119 eggs/female (Table 2). Mean fecundity was significantly higher on CMC-40 than on the other three entries (approximately 6–11 times higher). There were no significant differences among TST-18, TST-26, and MEcu-72.

**Figure 2.**
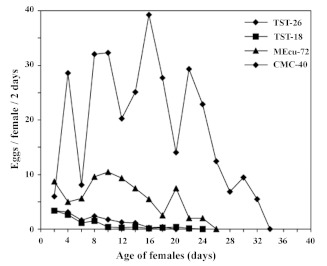
Oviposition curves of *Aleurotrachelus socialis* on *Manihot tristis* accessions, resistant MEcu-72 and susceptible CMC-40 genotypes. High quality figures are available online.

The *A. socialis* initiated oviposition on all the hosts during the first two days (Figure 2). Oviposition rates showed different patterns on the four entries, changing with age of the female. Oviposition rates decreased continually on TST-26 and TST-18, whereas they increased on MEcu-72 when females were between 18 and 22 days old, and then decreased. The oviposition rate for CMC-40 peaked on day 16 with 39 eggs/females/2days (Figure 2). The average oviposition rates on the *M. tristis* accessions (TST-26, TST-18) were comparable to the susceptible cultivar (Table 2). Fecundity values and oviposition rates were consistent. The TST-26 and TST18 accessions had the lowest values, suggesting that these hosts are less suitable for *A. socialis.* Based on these results, it can be concluded that when *A. socialis* females feed on *M. tristis,* they have the same longevity and reproduction rates as on MEcu-72, the most important cultivar resistant to *A. socialis.*

**Table 2.**
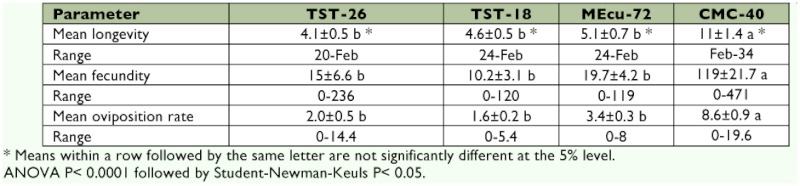
Longevity (± SE, days), fecundity (± SE, eggs/female) and oviposition (± SE, eggs/female/2 days) rates of *Aleurotrachelus socialis* female adults on *Manihot tristis* accessions, resistant MEcu-72 and susceptible CMC-40 (*n*=40) genotypes.

### Development time, survival of immature stages and proportion of females

The development time (egg-adult) of *A. socialis* was not significantly different among all hosts (Table 3). However, *A. socialis* took two more days to complete development on the *M. tristis* accessions and MEcu-72 than on CMC-40. The results found for the development time of *A. socialis* on CMC-40 in this study were similar to those reported by Gomez (2004) and Bellotti and Arias (2001), which were 32.8 and 32.1 days, respectively.

When *A. socialis* were maintained on the *M. tristis* accessions and the resistant cultivar MEcu-72, they had the lowest survival rates, which were not significantly different (χ^2^ = 52.53; df 3; p < 0.0001), but their means differed when compared with CMC-40 (Table 3). On CMC-40, the populations of *A. socialis* had the highest survival rate (0.93). It is important to note that immature stages of *A. socialis* on TST-26 and TST-18, and MEcu-72 had high survival rates (0.63 to 0.71). These values were lower than those obtained on MCol 2066 (0.87), the genotype used in Colombian plantations of commercial *M.* *esculenta* (Holguín et al. 2006), susceptible to *A. socialis.*

The proportion of *A. socialis* females was not affected by the potential resistance of the *M. tristis* accessions, being 1:1 on all the hosts (Table 3). The analysis of these results shows that the *M. tristis* accessions (TST-26 and TST-18) do have adverse effects on the survival rate of *A. socialis* without affecting the development time and proportion of females.

**Table 3.**
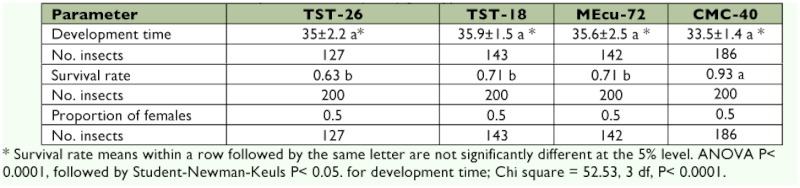
Development time (± SE, days), survival rate and proportion of *Aleurotrachelus socialis* females on *Manihot tristis* accessions, resistant MEcu-72 and susceptible CMC-40 (*n*=40) genotypes.

**Table 4.**
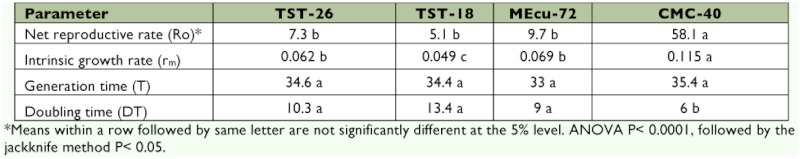
Demographic parameters of *Aleurotrachelus socialis* on *Manihot tristis* accessions, resistant MEcu-72 and susceptible CMC-40 genotypes.

### Demographic parameters

The demographic parameters calculated for the four hosts were different (Table 4). The results of the net reproduction rate (Ro) showed that on TST-26, TST-18, and MEcu72, an *A. socialis* female can have an average of from 5–10 female offspring in one generation, being not significantly different among them but different with respect to the susceptible genotype CMC-40, on which 58 female offspring were produced (p < 0.0001, followed by the jackknife method p < 0.05). These differences can be explained by the greater survival and fecundity of the females on CMC-40, which translated into a greater number of offspring at each age interval. The generation time (T) did not differ significantly among hosts (p < 0.0001, followed by the jackknife method p < 0.05). An *A. socialis* female takes an average of 33–35 days to complete one generation on the four hosts (Table 4).

These results are reflected in the *A. socialis* population's innate capacity for growth (r_m_). The analysis shows a significant decline in the potential for growth of the *A. socialis* population when fed on the two wild accessions (TST-18 and TST-26) and the resistant accession (MEcu72), being 47, 46, and 40% less, respectively, compared with the values observed on CMC-40 (p < 0.0001, followed by the jackknife method p < 0.05). Likewise, the time required for an *A. socialis* population to double its size was extended significantly by 3 to 7 days when fed on the *M. tristis* accessions and the resistant control MEcu72, compared with CMC-40 (p < 0.0001, followed by the jackknife method p < 0.05).

In conclusion, the results of this study reveal important levels of resistance to the whitefly, *A. socialis, on M. tristis* TST-26 and TST-18 accessions. This is due to the marked differences found for longevity and reproduction, which influenced and were consistent with the differences found in the net reproduction rate (Ro), intrinsic growth rate (r_m_), and population doubling time (DT). These findings suggested that factors related to fecundity, longevity, and subsequent effects on demography of *A. socialis* are probably responsible for the substantial differences found between the wild species accessions and CMC-40.

Taking into account the fact that recent research has shown that *M. tristis* has low populations of *M. tanajoa,* these results will enable breeding programs to incorporate resistance to this mite and whiteflies, the most important *M. esculenta* pests in the Americas and Africa (CIAT 2006), within elite lines in the near future. Furthermore, the new genomic tools, particularly molecular markers and marker-assisted selection, will make it possible to combine genes for resistance to *M. tanajoa* and *A. socialis* from a group of genes from the Neotropics in elite progenitors from Africa. The information generated in this study likely will contribute to the establishment of breeding programs that include the introgression of resistance traits found in the wild parent via backcrosses. Genomic tools, particularly molecular markers, make it possible to transfer genes for whitefly resistance from wild *Manihot* spp. to *M. esculenta.*

## Abbreviations

DAE: days after emergence;
DT: population doubling time;
Ro: net reproduction rate;
r_m_: intrinsic growth rate;
T: generation time

## References

[bibr01] Bailey H (1976). *Hortus Third. A Concise Dictionary of Plants Cultivated in the United States and Canada.*.

[bibr02] Balcazar VA (1997). *Desarrollos del cultivo de la yuca en Colombia.* Paper presented at the Global Cassava Development Strategy Progress Review Workshop, 10–11 June, Working, Doc. 5..

[bibr03] Bellotti AC, Riis L (1994). Cassava cyanogenic potential and resistance to pests and diseases.. *Acta Horticulturae*.

[bibr04] Bellotti AC, Van Schoonhoven A (1978). *Cassava Pests and Their Control.*.

[bibr05] Bellotti AC, Smith L, Lapointe SL (1999). Recent advances in cassava pest management.. *Annual Review of Entomology*.

[bibr06] Bellotti AC, Arias B (2001). Host plant resistance to whiteflies with emphasis on cassava as a case study.. *Crop Protection*.

[bibr07] Bellotti AC, Hillocks JM, Thresh JM, Bellotti AC (2002). Arthropod pests.. *Cassava: Biology, Production and Utilization*.

[bibr08] Brown JK, Frohlinch DR, Rossell RC (1995). The sweetpotato or silverleaf whiteflies: Biotypes of *Bemisia tabaci* or two species complex.. *Annual Review of Entomology*.

[bibr09] Burbano M, Carabalí A, Montoya-Lerma J, Bellotti AC (2003). Resistencia natural de especies silvestres *de Manihot* (Euphorbiaceae) a *Mononychellus tanajoa,* (Acariformes), *Aleurotrachelus socialis* y *Phenacoccus herreni* (Hemiptera).. *Revista Colombiana de Entomologia*.

[bibr10] Carey JR (1993). *Applied Demography for Biologists.*.

[bibr11] Ceballos H, La Yuca en Colombia y en el Mundo. (2002). La yuca en el 3° milenio.. Nuevas Perspectivas para un Cultivo Milenario.

[bibr12] CIAT. (2004). *Sustainable Integrated Management of Whiteflies Through Host Plant Resistance: Progress Report.*.

[bibr13] CIAT. (2006). *Manejo Integrado de Moscas Blancas Asociadas al Cultivo de la Yuca. Proyecto Manejo Integrado Sostenible de Moscas Blancas Como Plagas y Vectores de Virus en los Trópicos.*.

[bibr14] FAO/FIDA. (2000). La Economía Mundial de la Yuca: Hechos, Tendencias y Perspectivas..

[bibr15] Farras ARN (1994). Fluctuaçäo poblacional de *Aleurothrixus aepim* en mandioca, em São Miguel das Matas, Bahia. *Revista Brasileira*.. *Mandioca*.

[bibr16] Gomez MJ (2004). *Caracterización de la resistencia a la mosca blanca Aleurotrachelus socialis Bondar (Homoptera: Aleyrodidae) en genotipos de yuca (Manihot esculenta Crantz).*.

[bibr17] Henry G, Hershey C, Hillocks JM, Thresh JM, Bellotti AC (2002). Cassava in South America and the Caribbean.. *Cassava: Biology, Production and Utilization*.

[bibr18] Holguín CM, Carabalí A, Bellotti AC (2006). Tasa intrínseca de crecimiento de la población de *Aleurotrachelus socialis* Bondar en yuca *Manihot esculenta* Crantz.. *Revista Colombiana de Entomologia*.

[bibr19] Lee ET (1992). *Statistical Methods for Survival Data Analysis*, 2^nd^ ed..

[bibr20] Mara HNM, Luiz AJB, Campanhola C (2000). Statistical inference on associated fertility life tables parameters using jackknife technique: Computational aspects.. *Journal of Economic Entomology*.

[bibr21] Oliveira MRV, Henneberry TJ, Anderson P (2001). History, current status, and collaborative research projects for *Bemisia tabaci*.. *Crop Prot*.

[bibr22] Price P (1975). *Insect Ecology*..

[bibr23] SAS Institute. (1989). *SASISTAT User's Guide.*.

[bibr24] Vargas O, Bellotti AC (1981). Pérdidas en rendimiento causadas por moscas blancas en el cultivo de la yuca.. *Revista Colombiana de Entomologia*.

